# Use of Computational Modeling to Study Joint Degeneration: A Review

**DOI:** 10.3389/fbioe.2020.00093

**Published:** 2020-02-28

**Authors:** Satanik Mukherjee, Majid Nazemi, Ilse Jonkers, Liesbet Geris

**Affiliations:** ^1^Prometheus, Division of Skeletal Tissue Engineering, KU Leuven, Leuven, Belgium; ^2^Biomechanics Section, KU Leuven, Leuven, Belgium; ^3^GIGA in silico Medicine, University of Liège, Liège, Belgium; ^4^Human Movement Biomechanics Research Group, Department of Movement Sciences, KU Leuven, Leuven, Belgium

**Keywords:** *in silico* modeling, bone remodeling, cartilage degeneration, finite element modeling, gene regulatory network, data driven approach

## Abstract

Osteoarthritis (OA), a degenerative joint disease, is the most common chronic condition of the joints, which cannot be prevented effectively. Computational modeling of joint degradation allows to estimate the patient-specific progression of OA, which can aid clinicians to estimate the most suitable time window for surgical intervention in osteoarthritic patients. This paper gives an overview of the different approaches used to model different aspects of joint degeneration, thereby focusing mostly on the knee joint. The paper starts by discussing how OA affects the different components of the joint and how these are accounted for in the models. Subsequently, it discusses the different modeling approaches that can be used to answer questions related to OA etiology, progression and treatment. These models are ordered based on their underlying assumptions and technologies: musculoskeletal models, Finite Element models, (gene) regulatory models, multiscale models and data-driven models (artificial intelligence/machine learning). Finally, it is concluded that in the future, efforts should be made to integrate the different modeling techniques into a more robust computational framework that should not only be efficient to predict OA progression but also easily allow a patient’s individualized risk assessment as screening tool for use in clinical practice.

## Introduction

Osteoarthritis (OA), a degenerative joint disease, is the most common chronic condition of the joints, which cannot be prevented effectively. In Europe, over 100 million people suffer from arthritis. In the United States, nearly 2 million people under the age of 45 have symptomatic knee osteoarthritis (Deshpande et al., 2016). In 2013, total medical costs and earnings losses due to arthritis were $304 billion in the U.S. (about 1 percent of the U.S. gross domestic product for 2013) (Murphy et al., 2018). OA is a disease prevalent predominantly in the elderly, but it can also affect younger patients following injury, overuse (due to sports activities) and overweight. With an aging population coupled with other risk factors like obesity, the impact of OA on the society is suggested to only increase in the near future.

Contrary to earlier belief that OA is a cartilage disease, modern studies (Kuettner and Cole, 2005; Brandt et al., 2006; Lories and Luyten, 2011) suggest that it is a disease of the whole joint involving not only cartilage but also other joint constituents like the subchondral bone and bone marrow, menisci, ligaments and synovium. Indeed, initiation and progression of OA is characterized by changes in both the cartilage and the subchondral bone. In addition to the fact that the cartilage and subchondral bone are mechanically connected (Brown et al., 1984), there are evidences in literature that suggest the possibility of a biological crosstalk between the cartilage and bone, that is further increased as OA progresses (Berry et al., 1986; Imhof et al., 1999; Lyons et al., 2006). Thus, in order to study the initiation and progression of OA, the bone-cartilage unit (BCU) biomechanics need to be accounted for in the computational models.

It is also known that not all joints are equally prone to OA development. The joints that are most frequently affected by OA include the knee, hip, spine and the distal and proximal interphalangeal joints of the hand. Symptomatic OA less frequently occurs in the ankle, wrist, elbow, and shoulder joints (Kuettner and Cole, 2005). In these non-symptomatic joints, if degeneration of the articular cartilage occurs, it may be non-progressive, while in susceptible joints such a degeneration progresses to the OA state. For different joints, the kinematics and composition of the joint constituents are different, thereby resulting in different biomechanical joint environments. Also, the response of the chondrocytes in the cartilage and the underlying bone is different for different joints (Kuettner and Cole, 2005), which can also explain the susceptibility of certain joints to OA as compared to others.

It is already evident from the discussion above that in order to fully understand the onset and progression of OA, it is important to understand the biomechanical environment of anatomically complex joint structures like the knee joint. Since doing that experimentally is a challenging task, computational models can provide unique insights. Computational modeling of joint degradation can help to estimate the patient-specific progression of OA, which can aid clinicians to estimate a suitable time for surgical intervention in osteoarthritic patients. Likewise, they might also help to estimate whether certain physiotherapeutic strategies could be effective for arresting joint degradation. Furthermore, modeling approaches can also be used to test different hypotheses related to the underlying mechanisms of joint degeneration. However, the robustness of computational models in predicting joint degeneration will depend on whether they incorporate all the relevant degeneration mechanisms along different length scales and their interplay. Hence, verification and validation of the models with relevant experimental and clinical data is a crucial and important step within the computational modeling workflow. The variability in *in vivo* and *in vitro* experimental data inherent to biological specimens further emphasizes the need of population-wide model validation.

The objective of this review paper is to present an overview of different computational model formulations that have previously been used to study joint degradation. The advantages and shortcomings of each of these models, along with a comprehensive (yet non-exhaustive) set of examples of their use to study joint degeneration are discussed. The examples are focused on the knee joint since it is most prone to OA (Prieto-Alhambra et al., 2014) and the bulk of literature in computational modeling of joint degeneration is focused on the knee joint. Nevertheless, the concepts used for modeling the knee joint (including the damage mechanisms) can be easily extended to other joints and therefore more generalized conclusions can be drawn from these examples.

The paper is structured as follows: first, the different constituents of a joint are described as well as their changes with initiation and progression of OA. The following section describes the different computational model formulations that can be used to model joint degradation, including their advantages and disadvantages. The different computational models discussed include musculoskeletal models, finite element models (with different mechanisms of joint degeneration in the sub-sections), gene regulatory networks, multi-scale models, and data driven approaches. Thereafter the verification and validation aspects of these different computational models are discussed. Finally, some generalizable conclusions are drawn and suggestions for future work on further exploitation and integration of the different models are made.

## Joint Structure and Degenerative Changes

**FIGURE 1:**
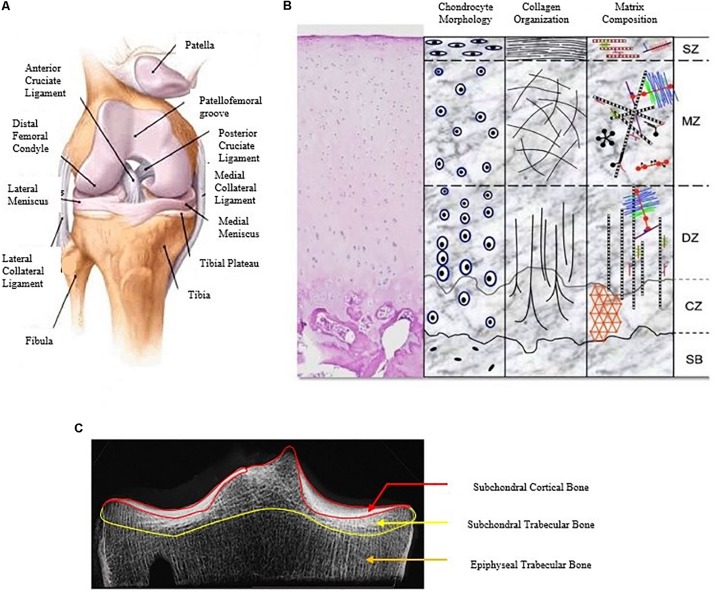
A detailed description of the human knee joint with the different constituents of the knee joint **(A)** (Islam, 2019), the different layers of articular cartilage showing variation in chondrocyte shape, collagen orientation, and matrix distribution **(B)** (Di Bella et al., 2015) and the different regions of the subchondral bone **(C)** (modified from Yamada et al., 2002).

**FIGURE 2:**
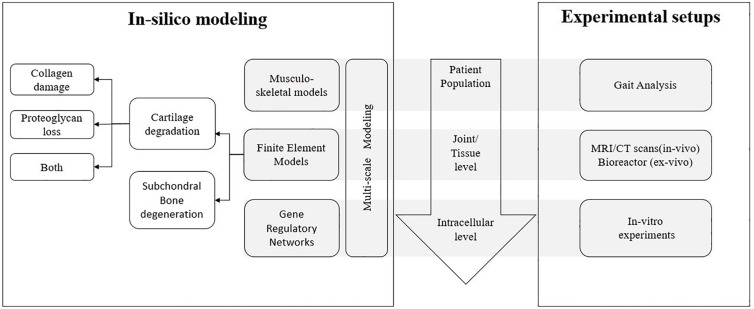
Overview of different aspects of joint degeneration with the different length scales involved and the link between *in silico* mechanistic modeling and corresponding experimental setups for each scale.

To develop a computational model of a human joint, three main inputs are required: (1) the anatomical properties of the joint (Figure 1), (2) the mechanical properties of the involved tissues, and (3) loading parameters (Erdemir et al., 2019). Obtaining the anatomical properties of the joint is the first step in the modeling pipeline, in order to represent the geometry and arrangement of different tissues involved. The anatomical features are virtually reconstructed from CT images or MRI scans by manual or (semi-)automated segmentation and consist of the bones (tibia, femur with or without patella), articular cartilage lining at the contact interfaces of the bones, ligaments holding the bones together and other contact structures; like the meniscus. Depending on the research question, the modeler has to make a choice on the fidelity and level of detail of the anatomy to be included in the model. The purpose of the following section is to briefly introduce the different components of the tibio-femoral joint; their specific constituents and their degenerative changes as well as their functional role.

### Articular Cartilage

Articular cartilage is the highly specialized connective tissue that covers the articulating ends of diarthrodial joints. Its primary function is to provide a lubricated, frictionless surface for smooth articulation and act as a cushion to distribute mechanical loads during contact between two joint surfaces. The articular joint surface consists of hyaline cartilage and is 2 to 4 mm thick in humans (Sophia Fox et al., 2009). Blood vessels, lymphatics and nerves are absent in the articular cartilage, and it is subject to high biomechanical loads. It is composed of a dense extracellular matrix (ECM) which is considered a biphasic material with a sparse distribution of cells called chondrocytes. Absence of vasculature limits its capacity for intrinsic healing and repair, therefore maintenance of its health is essential for joint health. The different constituents of articular cartilage are as shown in Figure 1B.

#### Water

Water is the primary constituent of articular cartilage, contributing up to 80% of its wet weight. A fraction of this water (approximately 30%) is contained in the intra-fibrillar space within the collagen, and a small fraction is contained in the intracellular spaces. The remaining water is contained in the voids of the porous ECM. The relative concentration of water increases from about 65% in the deep zone of cartilage to about 80% in the superficial zone (Sophia Fox et al., 2009). The transport and distribution of nutrients to chondrocytes in the cartilage, and lubrication at the cartilage surface is aided by the flow of water through the cartilage and across the articular surface respectively. Applying a pressure gradient across the tissue or compressing the solid matrix causes flow of the inter-fibrillar water through the ECM. Due to low permeability of articular cartilage, the ECM offers high frictional resistance against its flow. This frictional resistance to water flow through the matrix results in flow-dependent viscoelastic behavior of cartilage. The flow-dependent viscoelasticity and pore fluid (water) pressurization within the matrix are the two basic mechanisms by which articular cartilage can withstand very high physiological compressive loads (up to several times body weight).

##### Degenerative changes

With progression of OA and subsequent loss of proteoglycans and collagen fibrillation, several studies have reported an increase in the water content and hydraulic permeability of the articular cartilage (Setton et al., 1994; Wilson et al., 2005b; Mäkelä et al., 2015; Mononen et al., 2018). Mechanically, this increased permeability would reduce the dynamic stiffness of the cartilage due to a reduction in interstitial fluid pressurization and consequent deficiency of the load support mechanism in the degenerated cartilage.

#### Proteoglycans

Proteoglycans (PG) are an important constituent of the cartilage ECM which constitute approximately 30–35% of the dry weight of articular cartilage. They are large, complex biomolecules composed of a central protein core with negatively charged glycosaminoglycans (GAG) side chains (Wilson et al., 2005b). These negatively charged groups produce a high negative charge density which is quantified as the fixed charged density (FCD). Due to the FCD, the concentration of ions inside the cartilage is higher than that in the surrounding synovial fluid. This excess of ion particles in the tissue leads to a higher osmotic pressure in the tissue as compared to the surroundings. As a result of this osmotic pressure, swelling of the tissue occurs. This osmotic swelling is critical as it enhances the ability of cartilage to resist compressive loads.

##### Degenerative changes

Depletion of PGs are observed during the progression of OA (Wilson et al., 2005b; Saarakkala et al., 2010; Orozco et al., 2018), especially in the superficial zone of articular cartilage. In the literature, two mechanisms underlying PG depletion are proposed: (i) overloading of the cartilage, especially for post-traumatic OA, resulting in death of chondrocytes and release of inflammatory cytokines, thereby inhibiting production of PG and leading to PG depletion (Fick et al., 2016; Mononen et al., 2018); (ii) damage to the collagen fibrils during early stages of OA leading to increased fluid flow velocity across the surface, thereby promoting loss of matrix fragments and loss of superficial PG (Thibault et al., 2002; Rieppo et al., 2003). Mechanically, PG damage will not only reduce the matrix stiffness but also increase the tissue permeability thereby reducing its dynamic stiffness. In addition, PG loss reduces the cartilage FCD and reduces the consequent swelling pressure.

#### Collagen

Collagen is the most predominant structural macromolecule in ECM, constituting about 60% of the cartilage’s dry weight. Type II collagen constitutes 90 to 95% of the collagen in ECM and forms fibrils and fibers intertwined with proteoglycan aggregates (Sophia Fox et al., 2009). Collagen fibers offer resistance to tension. Hence, they provide resistance against swelling and tensile strains, but they do not offer significant resistance to compression. In the deep zone of cartilage, the collagen fibrils are perpendicular to the cartilage surface whereas in the superficial zone, they are parallel to the surface, thereby resisting shear deformation of the surface.

##### Degenerative changes

Damage to the collagen network is one of the early signs of OA and has been recorded in many studies (Wilson et al., 2006b; Mononen et al., 2012; Hosseini et al., 2014). This has also been associated to the initial cartilage swelling observed in early OA (Bank et al., 2000). Collagen damage furthermore triggers loss of PGs and loss of tissue hydration as described in the previous section. Mechanically, damage to the collagen network will lead to a significant reduction in the tensile stiffness of the cartilage and enhanced swelling, since the collagen network helps constraining the swelling of the tissue.

#### Chondrocytes

Chondrocytes are the cells that reside in the articular cartilage. They are highly specialized cells which are metabolically active. They play a pivotal role in the development, maintenance, and repair of the ECM as well as its degeneration (in case of OA). Chondrocytes constitute only about 2% of the total volume of articular cartilage. The shape, number, and size of chondrocytes vary considerably across the thickness of the articular cartilage. The chondrocytes in the superficial zone of cartilage are flatter in shape and smaller in size and generally have a greater density than those present in the deeper zones in the matrix. They can sense and respond to a variety of stimuli, such as growth factors, mechanical loads, and hydrostatic pressures. They do not easily proliferate in cartilage, which limits the healing capacity of the cartilage post-injury or damage. An optimal chemical and mechanical environment is therefore critical to the survival of chondrocytes.

##### Degenerative changes

In OA, the balance between the anabolic and catabolic factors is disturbed, with an increase in the latter. During the early stages of OA, the levels of PG and collagen synthesis are largely increased (Lippiello et al., 1977; Clouet et al., 2009) to counterbalance the upregulated catabolic processes such as the increased production of matrix degrading metalloproteinases (Okada et al., 1992; Moldovan et al., 1997). In further stages of OA, chondrocyte apoptosis leads to the degradation of cartilage (Chen et al., 2003; Kim and Blanco, 2007). Chondrocytes in OA have been shown to express hypertrophy markers such as type X collagen and MMP13 (Kirsch and von der Mark, 1992; Nurminskaya and Linsenmayer, 1996; Alvarez et al., 2000). It is therefore hypothesized in some studies that during OA, articular chondrocytes lose their stable differentiated phenotype and behave like terminal differentiating chondrocytes as found in the growth plate of long bones, expressing hypertrophy-like changes (von der Mark et al., 1992; Dreier, 2010; van der Kraan and van den Berg, 2012).

### Calcified Cartilage

The zone of calcified cartilage (ZCC) acts as a transition zone from the softer cartilage to the stiffer sub-chondral bone region. The ZCC serves the important task of transmitting forces between the softer cartilage and the stiffer bone, thereby distributing the stresses across the bone-cartilage interface. The ZCC has intermediate stiffness with previous studies showing that the stiffness of the ZCC is about 100 times the overlying hyaline cartilage and 1/10 times the underlying subchondral bone (Hargrave-Thomas et al., 2015). Apart from providing an intermediate stiffness, the ZCC is believed to act as a biochemical barrier preventing molecular exchange between the articular cartilage and the underlying bone marrow.

#### Degenerative changes

In OA, the ZCC is thought to cause thinning of the overlying articular cartilage by mineralization and advancement of the tidemark toward the articular cartilage layer (Oegema et al., 1996, 1997). Thinning of articular cartilage would then increase forces on the bone and thereby promote further degeneration. Some studies also suggests that vessels and channels that connect the subchondral bone with the calcified and the uncalcified cartilage become more abundant in the cartilage of patients with OA as compared with that of healthy individuals, thereby increasing the molecular cross-talk between the bone and cartilage cells (Thambyah and Broom, 2007; Walsh et al., 2010; Lories and Luyten, 2011).

### Subchondral Bone

There are different layers of bone beneath cartilage, the subchondral cortical bone, the subchondral trabecular bone, and the epiphyseal trabecular bone which are collectively regarded as the subchondral bone (Figure 1C). The subchondral cortical bone or subchondral cortical plate is a thin layer of compact bone beneath the calcified cartilage, separated from it by a thin layer of ductile material called the cement line, and is composed of about around 48% mineral, 31% organic matter, and 21% water in a healthy samples (Li and Aspden, 1997b). The thickness of subchondral cortical end-plate varies both within a sample and between different samples and could additionally be affected by OA. A range between 0.1 and 4.8 mm has been reported in the literature (Clark and Huber, 1990; Grynpas et al., 1991; Milz and Putz, 1994; Yamada et al., 2002; Buckland-Wright, 2004), with increased thickness at the late stages of OA (subchondral sclerosis) (Buckland-Wright, 2004; Goldring, 2012; Li et al., 2013; Aho et al., 2017). Likewise, a range of elastic moduli from 0.6 to 20.0 GPa has been reported for the subchondral cortical bone (Choi et al., 1990; Li and Aspden, 1997b).

The subchondral trabecular bone is a layer of cancellous bone beneath the subchondral cortical bone. Subchondral trabecular bone layer thickness varies within and between samples with reported values ranging from 1 to 4 mm (Johnston et al., 2011). The apparent elastic modulus has been reported to vary between 6 and 1670 MPa (Bentzen et al., 1987; Finlay et al., 1989; Zysset et al., 1994; Day et al., 2001; Amini, 2013). Under normal circumstances at the proximal tibia, mean bone volume fraction, trabecular thickness, trabecular spacing, and trabecular number are 0.296, 146, 392 μm and 2.07/mm, respectively (Kamibayashi et al., 1995).

The epiphyseal trabecular bone is a layer of cancellous bone which extends from beneath the subchondral trabecular bone to the growth plate (Figure 1C). Compared to subchondral trabecular bone, epiphyseal trabecular bone has lower mean bone volume fraction (Kamibayashi et al., 1995) and thinner trabeculae (Kamibayashi et al., 1995). A range of elastic moduli from 10 to 2770 MPa has been reported for the epiphyseal trabecular bone (Keyak et al., 1994; Zysset et al., 1994; Morgan et al., 2003; Amini, 2013).

#### Degenerative changes

The subchondral cortical bone’s composition is influenced by OA, leading to reduced mineral content and increased water content (Li and Aspden, 1997b). Subchondral bone tissue stiffness generally decreases with OA (Day et al., 2001; Coats et al., 2003; Dall’Ara et al., 2011) which might be attributed to the lower mineral content (hypo mineralization) (Li and Aspden, 1997a, b; Chappard et al., 2006; Cox et al., 2012). Increased subchondral trabecular bone volume fraction and thickness, and decreased trabecular spacing and degree of anisotropy have, however, been observed in the later stages of OA (Ding et al., 2001, 2003; Chappard et al., 2006; Cox et al., 2012). Animal studies have revealed decreased epiphyseal trabecular bone volume fraction with OA progression (Dedrick et al., 1993). It is known that the microstructure and tissue density affects the apparent density and tissue modulus and microstructure affects the apparent modulus. Therefore, alterations of subchondral bone apparent density and modulus have been observed with OA progression. Various cadaveric and animal studies have shown decreased apparent density and modulus at early stages of OA (Ding et al., 1998, 2001; Batiste et al., 2004; Intema et al., 2010) which is thought to be due to elevated bone remodeling evidenced by vascular invasion at the cartilage-subchondral bone junction, and subchondral plate thinning and increased porosity (Burr and Schaffler, 1997; Intema et al., 2010). At the late stages of OA, however, apparent density and modulus have been reported to be significantly higher compared to normal subchondral bone (Li and Aspden, 1997a, b; Johnston et al., 2009). The structural stiffness at the subchondral surface is a result of the specific spatial distribution of the apparent modulus and is thus expected to alter as OA progresses. Altered structural stiffness at the subchondral surface as the elastic foundation supporting the overlaying cartilage may influence the distribution of stress and strain in the cartilage leading to accelerated degeneration (Radin et al., 1970, 1972; Radin and Rose, 1986).

### Ligaments

The primary function of the ligaments is to tie the bones of the joint together, thereby stabilizing its motions. Any injury or damage to the ligaments are known to trigger abnormal joint kinetics and kinematics, which may contribute to joint degeneration due to excessive shear forces (Shim et al., 2011; Simon et al., 2015).

#### Degenerative changes

Injury to the anterior cruciate ligament (ACL) of the knee is known to increase the risk of osteoarthritis. ACL deficiency results in disruption of the normal physiological knee kinematics especially inducing increased internal tibial rotation and increased anterior tibial translation upon flexion. This has been associated with increased mean contact stresses in the posterior medial and lateral compartments of the knee joint under anterior and rotational loading which may trigger OA (Simon et al., 2015).

### Meniscus

Menisci are located between the tibia and femur bone. They are two crescent-shaped pads of cartilage that evenly transfer load across the joint, absorb shocks during dynamic movement, and lubricate and help to stabilize the joint.

#### Degenerative changes

Injury, degeneration, or surgical removal (meniscectomy) of the entire or part of the meniscus is known to increase the risk of developing knee OA (Englund and Lohmander, 2006).

## Use of Computational Models to Study Joint Degeneration

Computational modeling of joint degeneration can be useful to predict the initiation and progression of degradation in the different components of the joint, thereby aiding clinicians in timely intervention (e.g., weight loss, surgery, rehabilitation). Computational models can range from joint level musculo-skeletal models that evaluate abnormal whole joint loading conditions that may lead to joint degradation; finite element models which can evaluate tissue loading and predict their degeneration in response to mechanical loading and gene regulatory network models which can predict the fate of chondrocytes when subjected to abnormal mechanical or biochemical stimuli, thereby predicting the cellular processes underlying joint degradation. The choice of the specific modeling strategy depends on the specific research question that is to be answered. It is also evident from the discussion above that degradation happens due to interplay of factors which are active at different length scales. Therefore, multiscale modeling can be useful to combine the different length scales and to develop a more robust (computational) model of joint degeneration.

In this section, we will discuss the different computational modeling strategies used to date to study joint degeneration, a flowchart of which is shown in Figure 2. This section is structured as follows: firstly, musculoskeletal models are discussed with their advantages and disadvantages, followed by some examples from literature which involves the use of musculoskeletal models to study joint degeneration. Secondly, finite element models are discussed with their advantages and disadvantages. The examples of finite element models have been grouped based on the component of the joint that is being modeled for degeneration. Thirdly, gene regulatory network based models are discussed with their advantages and disadvantages, followed by some examples from literature which involves its use to study joint degeneration. Fourthly, multiscale models are discussed with their advantages and disadvantages, followed by some examples from literature which involves the use of multiscale models in the perspective of joint degeneration. Finally, the use of data driven approaches to study joint degeneration is discussed with its advantages and disadvantages and some examples.

### Musculoskeletal Models

Joint degradation is generally associated with altered gait patterns of the patient (Li et al., 2013; Farrokhi et al., 2015; Favre and Jolles, 2016). This altered gait kinematics and kinetics then further increase the joint contact forces which will further promote the degeneration (Baliunas et al., 2002; Meireles et al., 2017; Richards et al., 2018). In this context, musculoskeletal computational models, with inputs from experimental motion and ground reaction force capture, can be used to quantify the joint contact forces and consequent pressures, which identify the presence of excessive loading at joint level and therefore predict the risk of damage initiation or progression.

#### Advantages and Limitations

Musculoskeletal modeling provides a non-invasive way to calculate internal biomechanical loads from experimentally measured 3D body motions and contact forces in a wide range of conditions. These joint contact forces give an estimate on the presence of excessive loading and therefore the risk of damage initiation or progression. However, as these models only provide an overall estimate of joint loading, they cannot identify areas of damage initiation, nor progression as they do not quantify tissue-specific stresses and strains. The determination of the properties of a musculoskeletal model (such as bone lengths, muscle attachment sites, joint centers, etc.) is typically based on reference values from anatomic and cadaveric studies. This result in a generic musculoskeletal model which needs to be adapted and scaled to make it patient-specific. However, these linear scaling approaches can never mimic subject-specific geometries and therefore do not allow subject-specific predictions, thereby inherently limiting the use of this approach. On the other hand, medical imaging-based workflows using CT- or MR images have been developed allowing the introduction of patient-specific geometries in the musculoskeletal model (Scheys et al., 2008). Although the geometrical precision of these models clearly outperforms scaled generic musculoskeletal models, their application is usually cumbersome, expensive, time-consuming, dependent on a skilled operator and is therefore less feasible for large-scale studies. More recently, the use of population-based approaches (such as the MAP client) have been used to personalize generic musculoskeletal muscles, using a sparse data set of personalized geometrical features (e.g., from Killen et al., 2018).

#### Examples

It has been shown by Richards et al. (2018) that for patients with medial knee OA, the knee adduction moment (KAM) is a strong predictor of the medial knee contact force (mKCF) at the first peak during normal walking. It was also reported in this study that walking with toe-in or wide-steps gait, modified the first peak KAM, but no reduction in mKCF was observed. However, the ratio of mKCF to total KCF, which represents the distribution of the loading, was reduced. This study demonstrated the potential of musculoskeletal models to not only identify markers that increase joint loading, thereby triggering more degeneration, but also to define modified gait strategies to reduce the joint loading in an already degenerated joint. In Meireles et al. (2017) it was shown that the medial-lateral force and contact pressure distributions were already altered in early stages of medial knee OA during normal gait. No such trend was observed for a step-up-and-over motion.

### Finite Element Models

The finite element method (FEM) has been used widely to calculate stresses and strains in the different joint tissues (Mononen et al., 2012, 2015; Halonen et al., 2013, 2014; Kazemi et al., 2013; Erdemir et al., 2019). Since onset and progression of joint degradation are hypothesized to be a function of excessive tissue stresses and strains, finite element models can be particularly effective in calculating the mechanical environment in the tissues which are then thought to trigger degeneration. The steps that are generally followed to develop FE based models of joint degeneration are as follows.

(a) Representation of appropriate geometry of the joint and material properties of the constituent tissues.
(b) Input boundary conditions in terms of joint motions, loads and constraints, often derived from a musculoskeletal modeling workflow.
(c) Identification of output modeling parameters relevant to joint degeneration.
(d) Formulation of adaptive -degeneration- algorithms which relate the output parameters from the FE model to degeneration of the joint constituents.

Using such a workflow, the initiation and progression of degradation of knee joint structures has been quantified based on an iterative evaluation of the finite element model-based stresses or strains and joint degeneration algorithms (Hosseini et al., 2014; Mononen et al., 2016; Orozco et al., 2018).

#### Advantages and Limitations

With advancement in imaging technologies, finite element models typically aim to incorporate the complex geometry of the different joint tissues as well as realistic material definitions. This increases the biofidelity of the FE models to emulate the *in vivo* mechanics of the joints. However, it is nearly impossible to obtain an accurate representation of the joint geometry due to image resolution, inaccuracies during the segmentation process as well as smoothing techniques commonly used to ensure better contact convergence in the joint. In addition, and comparable to our discussion on musculoskeletal modeling, manual segmentation of the geometry from imaging data to build a patient-specific model is a time consuming task, and hence is not suitable to be integrated in a routine manner in the clinic. There have been quite a few approaches to automate the process of segmentation (Marstal et al., 2011; Lee et al., 2014; Dam et al., 2015; Ye et al., 2015; Bonaretti et al., 2019). However, most approaches are directed at automatic segmentation of the articular cartilage, while omitting the other significant tissues of the joint such as the ligaments or the meniscus [except for Dam et al. (2015), where meniscus was included], which will restrict the accuracy of the *in silico* models to predict the *in vivo* conditions. Very sophisticated constitutive models have been developed for cartilage and other joint constituents (Wilson et al., 2005a; Julkunen et al., 2013; Mononen et al., 2015), but obtaining sufficient experimental data to estimate the parameters for these models is quite a challenging task. In most cases, these parameters are estimated from *in vitro* experiments of cadavers, bovine or porcine joints, since determination of patient specific material properties is not always possible. Given that there is a huge variability in the material properties of biological samples, it leads to departure from patient-specificity of the developed models. Furthermore, limited tools are currently under development that allow non-invasive characterization of the material properties of the joint complex, which is necessary to develop FE models. Although MRI-based relaxometry is a promising technique (MacKay et al., 2018), it merely is a qualitative more than quantitative imaging modality whose role in multi-scale modeling to predict patient-specific OA progression needs to be further confirmed (Julkunen et al., 2013). Also, the constitutive models for cartilage tissue do not consider physical phenomena like buckling of the collagen fibrils, fibril–fibril interactions and width of the collagen fibrils, nor fluid-pressurization-induced lubrication effects (Julkunen et al., 2013). In addition, there are diverging opinions in literature on the FE output parameters to be used for the degeneration algorithms for the different joint constituents. Therefore, in the following examples, existing literature covering a variety of output parameters relevant for joint degeneration is discussed. Verification and validation of the degeneration algorithms with experimental data is also a challenging task and will be discussed in more details in Section “Verification, Validation, and Uncertainty Quantification of Computational Models.”

#### Examples

Since there are different models that exist in literature for the degradation of the different joint constituents, the examples have been grouped based on the constituent for which degeneration is being modeled using FEM. Hence, the names of the following subsections will be based on the constituent of the joint, keeping in mind that all the subsection involve the use of FEM as the common computational method (see breakdown in Figure 2).

##### FE-models of articular cartilage degeneration in OA

Articular cartilage is one of the primary load-bearing joint structures which may become damaged when cartilage is subjected to excessive mechanical loading, and such damage is likely to progress into OA. Early signs of OA include loss of PG and roughening of the cartilage surface. In the later stages, fibrillation of the cartilage occurs, with cracks on the surface penetrating deeper into the tissue (Chen and Broom, 1998; Hosseini et al., 2014). State of the art computational models include the different constituents of articular cartilage and account for fundamental mechanical properties such as collagen fiber reinforcement with physiological organization of the collagen structure, ground substance (ground matrix) stiffness and tissue swelling due to PGs, while accounting for a depth dependent collagen and PG density (Wilson et al., 2005a). In consequence, their effects on the mechanical response of the cartilage can be numerically studied. This way, a holistic understanding of cartilage degeneration can be achieved, as damage in the individual components of cartilage, i.e., softening in the ground substance and damage to the collagen network, and the possible interaction between these two can be studied. As it is challenging to explore these effects experimentally, computational models provide unique insights. In the following subsections, FE models for the degeneration of the different constituents of articular cartilage will be discussed.

*FE-modeling of proteoglycan degeneration:* Loss of proteoglycans is one of the signs of early OA. Proteoglycans being the fixed charge carriers in cartilage, the degeneration of PGs has a two-fold effect: reducing the cartilage’s compressive properties (due to increased permeability) and fixed charge density (FCD), resulting in reduced osmotic swelling pressure. In the paper (Orozco et al., 2018), FCD loss was modeled in lesioned cartilage disks under dynamic loading using a degeneration algorithm driven by fluid velocity, maximum shear strain and deviatoric strain separately. The fluid velocity driven algorithm was found to be most effective in predicting FCD loss when compared to experimental results. This 2D model incorporated only loss of FCD as a result of PG loss. No softening of the cartilage ground substance due to PG loss was considered, which is a shortcoming of this study. Another study (Eskelinen et al., 2019) involving a 3D FE model of a lesioned cartilage disk, used similar degeneration algorithms, however with different parameters driving the degeneration algorithm, more specifically axial strain, shear strain, octahedral shear strain, maximum shear strain, minimum principal strain and maximum principal stress. A maximum shear strain-driven algorithm resulted in the maximum degeneration of fixed charges around the lesion. It was also observed that the choice of the degeneration thresholds for each of the cases as well as the choice of the loading regime largely influenced the degeneration of fixed charges around the lesion. Also, the collagen fibril stiffness was reduced and the permeability increased to model a more realistic case of loss of fixed charges, further increasing the FCD degeneration.

It must be noted that both these models incorporate local damage theories, where the damage at an integration point is determined by the stresses or strains at that point only. This local damage theory could result in localization of damage which results in concentration of all damage in a vanishing volume. Based on past literature on damage mechanics (Pijaudier-Cabot and Bažant, 1987; de Vree et al., 1995), this could cause an extreme brittle behavior of the material, with the material failing at almost zero energy (Peerlings et al., 2002). In context of cartilage damage, it would mean that damage to cartilage occurs catastrophically as compared to a more gradual damage in physiological case. The solution to this problem would be to use non-local damage theories, such as the one used by Párraga Quiroga et al. (2017).

**FIGURE 3:**
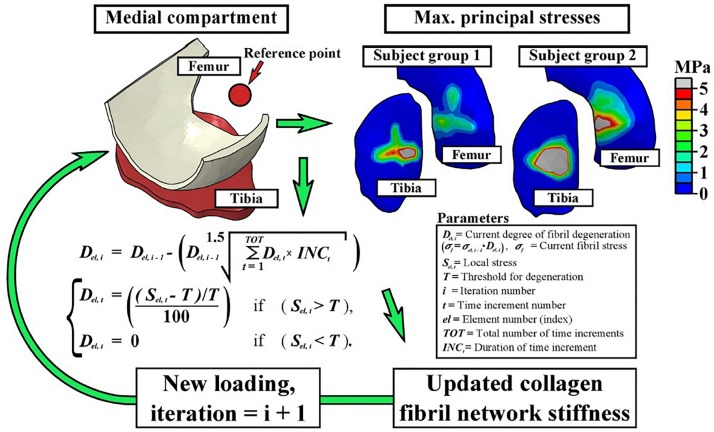
The fibril degeneration algorithm (shown in the left side) was based on excessive maximum principal stresses in the medial compartment of the knee joint. The stress distributions on the right are obtained at the first peak loading force of the stance phase of gait. The model was run iteratively to simulate gradual degeneration of the collagen fibril network (Mononen et al., 2016).

*FE-modeling of collagen fiber degeneration:* During the initiation phase of OA, an increase in the fibrillation of the collagen fibril network is observed. Hence, modeling the degradation of the collagen fibrils in articular cartilage as a result of repeated mechanical loading is an important step in the study of cartilage degeneration. In one study (Mononen et al., 2016), a collagen degeneration algorithm was implemented for the first time in a human knee joint model, thereby representing damage accumulation in overweight patients during physiological gait loading. In this algorithm, the stiffness of the local collagen fibril network was reduced when the maximum principal stress in the tissue exceeded a threshold value based on a chosen criteria as shown in Figure 3. By performing 100 iterative simulations, the authors observed that fibril degeneration in the medial tibial cartilage was more pronounced for obese subjects compared to healthy humans. These results also confirmed based on an experimental 4 year follow-up in patients. As such, this study was one of the first in its kind demonstrating the huge potential for simulation of patient-specific cartilage degeneration. In another follow-up study (Liukkonen et al., 2017), the same degeneration algorithm was used with patient specific FE models representative of different grades of OA involvement. The algorithm was able to predict OA progression when compared to the experimental follow-up data for different subjects. Maximum degeneration and degenerated volumes within the cartilage were found to be significantly higher in OA subjects as compared to healthy subjects.

Different opinions exist in literature regarding the physical parameters influencing the degeneration of collagen fibrils. In the aforementioned study (Mononen et al., 2016), maximum principal stress in the tissue was considered to influence collagen damage, whereas in another study (Wilson et al., 2006b), it was shown that for relatively thick cartilage samples, collagen damage is caused by excessive shear strains along the collagen fibrils, whereas in thin samples, collagen damage is caused by both excessive shear strains along the collagen fibrils and collagen fibril tensile strains, at distinct locations. It was concluded that cartilage damage starts due to excessive shear strain along collagen fibrils and damage due to excessive fibrils strains occur at higher loads.

Apart from degradation of collagen fibrils, collagen network disorganization could progress following cartilage injury, which can lead to further cartilage degeneration (Ferizi et al., 2017). Past works on computational modeling of collagen network disorganization in injured articular cartilage include studies by Wilson et al. (2006a) and Tanska et al. (2018). Tanska et al. (2018) concluded that when the collagen reorientation algorithm was based on both tensile tissue stress or strain and tensile collagen fibril strain, substantial collagen reorientation was predicted locally near a cartilage defect and particularly at the cartilage–bone interface.

**FIGURE 4:**
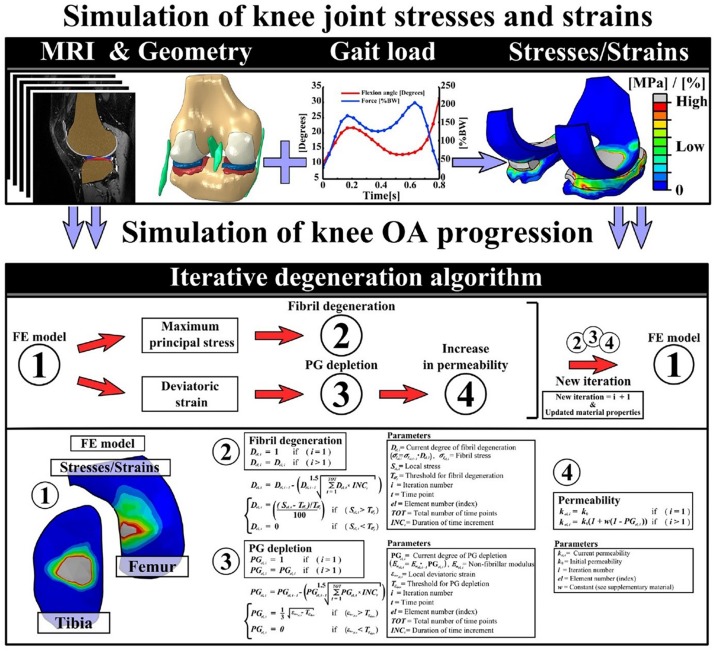
This figure presents the detailed workflow followed to implement an iterative degeneration algorithm. The top row describes the steps followed to develop the FE model to simulate knee joint stresses and strains. The middle row shows the different components of articular cartilage that are considered to be degenerating in the algorithm. The last row presents the mathematical formulations used in the algorithm to model collagen fibril degeneration, proteoglycan depletion, and increase of permeability of the cartilage due to excessive stresses and strains (Mononen et al., 2018).

*FE-modeling of combined collagen fibril and proteoglycan degeneration:* since the initiation and progression of OA is characterized by collagen fibrillation, and an increase in fluid fraction and proteoglycan depletion (Andriacchi et al., 2004; Wilson et al., 2005b; Orozco et al., 2018), there is a need to integrate all these mechanisms while developing a robust computational model of cartilage degeneration. In this context, a study by Hosseini et al. (2014) aimed to understand the interaction between ground substance softening and fibril degeneration and their combined effect on overall cartilage degeneration. For the damage model, it was assumed that excess deviatoric strain in the ground substance beyond a particular threshold would lead to softening of the matrix. For the fibril network, it was assumed that strain in the direction of the fibers exceeding a threshold would lead to fiber softening. It was observed that under applied indentation loading, ground substance softening developed over a larger area than collagen damage. Damage in the ground substance affected cartilage softening earlier and to a greater extent than collagen damage. In a similar study by Mononen et al. (2018), degeneration of collagen fibrils and PG content, combined with an increase in cartilage permeability of cartilage was modeled. While the degeneration of collagen fibrils was triggered by excessive and cumulative tensile stresses, degeneration of PG’s was modeled by a reduction in the ground substance modulus due to excessive deviatoric strains. A decrease in the proteoglycan content was equated with an increase in the tissue permeability, leading to more fluid flow. The detailed schematics of the study is shown in Figure 4.

In another study Párraga Quiroga et al. (2017) investigated the effect of strain rate on cartilage damage. Given that cartilage is poro-viscoelastic, strain-rate dependent effects are indeed expected. The authors observed that with increasing strain rate collagen damage increased, whereas damage in the non-fibrillar matrix decreased. It must be noted that this study used the same damage evolution as in Hosseini et al. (2014). However, they incorporated a non-local damage theory, where, instead of using the local strain in each integration point for damage evaluation, a strain averaged over a specific volume around the integration point was used. The results showed that the local damage approaches result in larger damage areas, localized to specific elements, whereas the non-local approach shows lower damage values as a result of the weight-averaging damage based on Gauss function.

Alternatively, phenomenological models have also been developed to model the degradation of cartilage (Landinez-Parra et al., 2011; Argatov and Mishuris, 2015; Men et al., 2017). In one of these studies (Landinez-Parra et al., 2011), a phenomenological mathematical damage model caused by mechanical action was developed. The model considered tissue failure as a result of chondrocyte death and matrix loss. It took into consideration different factors modifying fatigue resistance such as age, body mass index (BMI) and metabolic activity. These FE simulations were able to predict tissue failure at different loading frequencies, variations in damage magnitude and also different damage sites.

##### FE models of subchondral bone changes in OA

Simplistic FE bone models have been developed to evaluate the structural role of subchondral bone in OA progression (Little et al., 1986; Amini et al., 2015). Such models, however, disregard the intra-specimen spatial distribution of material properties and also the subtle geometric difference between subjects. QCT-FE models of subchondral bone have recently been developed to monitor alterations of local structural stiffness at the subchondral surface non-invasively and subject-specifically (Nazemi et al., 2015, 2017). In such models, the QCT provides information regarding the geometry and density of the imaged bone whereas FE calculates the integral contribution of all factors involved in the structural stiffness sensed at the subchondral surface. More advanced imaging techniques, e.g., MRI/CT imaging using contrast agents (Matzat et al., 2014), could help to construct subject-specific FE models of the cartilage-subchondral bone complex.

##### Coupled FE models of cartilage degeneration and subchondral bone remodeling in OA

Since the degeneration of cartilage and remodeling of the subchondral bone progress hand-in-hand in case of OA (Lories and Luyten, 2011), both the processes must be coupled in computational models for a more accurate (patho)physiological description. In a study (Stender et al., 2016), the BCU was modeled consisting of the articular cartilage, calcified cartilage, subchondral cortical bone and subchondral trabecular bone, however assuming a simple geometry. Bone remodeling was assumed to occur in the subchondral cortical and trabecular bone, and damage to articular cartilage could occur either via damage to collagen fibrils or degradation of GAGs. Results from spherical indentation on the articular cartilage surface of the BCU indicated that damage to articular cartilage occurs at the articular surface. Furthermore, bone remodeling was also predicted to occur with a net stiffening of the subchondral bone plate. In another study involving poro-elastic properties of BCU (Stender et al., 2017), it was observed that the permeability of the articular cartilage governs the poro-mechanical response of the BCU while the permeability of calcified tissues exerts no appreciable effect on the force-indentation response of the BCU. With OA permeability properties for the bone and cartilage, higher fluid velocities were observed. *In vivo*, this phenomenon would likely lead to chondrocyte death, tissue remodeling, alterations in joint lubrication, and the progression of osteoarthritis.

### Gene Regulatory Network Models

Computer models can also provide a formal framework to study the dynamics of genetic programs happening inside a cell. Such computational approaches are highly relevant for systems biology, a field that has gained quite some importance in the field of tissue engineering and regenerative medicine over the last years. One family of models, the (gene) regulatory network (G)RN models, can be particularly interesting to decipher signaling and cell responses implied in cell fate decisions. Since the complex interplay of different factors present in signaling networks can impossibly be dealt with by human intuition, *in silico* models involving formal computer languages can provide unique insights. There exist quite some examples in literature (Woolf et al., 2005; Xia et al., 2006; Aldridge et al., 2009; Saez-Rodriguez et al., 2009) where *in silico* models were successfully used to decipher biological complexity and give new biological insights.

**FIGURE 5:**
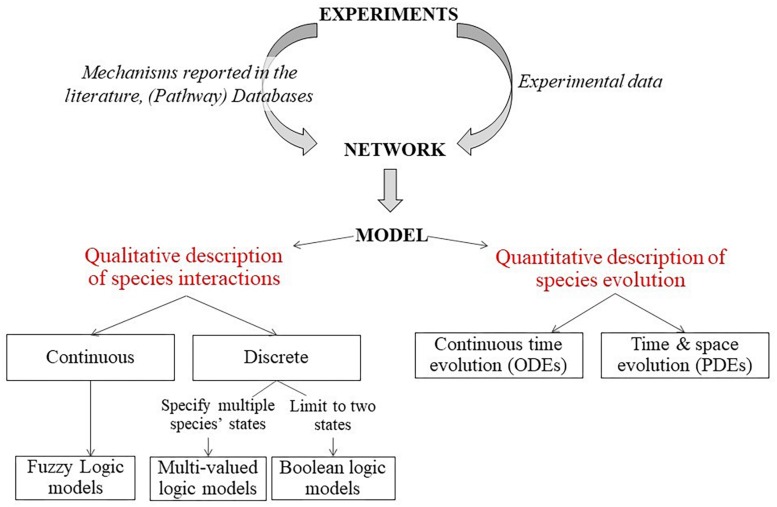
Description of modeling framework for regulatory networks. Firstly, a static network graph is generated from experimental data and mechanisms reported in literature. Then, various modeling approaches can be used to simulate the temporal evolution of the components of the network. Quantitative models use ODE to describe the temporal evolution of species (cell density etc.) and can also include spatial resolution by using partial differential equations. Qualitative models on the other hand use logical statements to describe the evolution of species (Lesage et al., 2018).

The overview of different methods that can be used to generate the (G)RN models are shown in Figure 5 (Lesage et al., 2018). The first step is to generate a network graph, which provides a static (unchanging) representation of the biological processes under study. This network graph can be either inferred directly from the experimental data (data-driven approach) or derived from mechanisms and pathways already available in the literature (knowledge-based approach). Once a network is established, a dynamic analysis is performed to simulate the temporal evolution of the different network elements under specific conditions and to study the possible outcomes (stable states) of the established network. Various modeling approaches can be used to simulate the evolution of the network components over time. Quantitative models use ordinary differential equations (ODEs) to describe the temporal evolution of species (cell density, etc.) and can also include spatial resolution by using partial differential equations. Qualitative models on the other hand use logical statements to describe the evolution of species (Morris et al., 2010).

#### Advantages and Limitations

Regulatory networks can be useful to study the molecular mechanisms of joint degeneration. In the adult stage, the chondrocytes in the articular cartilage remain in a stable phenotype characterized by a low rate of proliferation and the production of ECM rich in Col-II and Aggrecan. However, some degenerative diseases such as OA may lead to dysregulation of the stable cartilage phenotype, modifying the chondrocyte’s proliferation rate and triggering its switch toward hypertrophy, thereby leading to abnormal ossification of the joints. (G)RN models of stable chondrocytes can be useful to study the intracellular activation of different pathways following mechanical and chemical signals, thereby leading to cell phenotype changes due to cell dedifferentiation, inducing OA. Since these regulatory network graphs are sometimes constructed on the basis of experimental results published in literature (knowledge-based models), conflicting opinions in literature can lead to problems in decision making from the perspective of the model developer. Additionally, most of these (G) RN models are either qualitative or semi-quantitative in nature (Kerkhofs and Geris, 2015; Kerkhofs et al., 2016), which makes it difficult to be integrated with quantitative methods like the FEM. Also, the ability for quantitative model predictions is limited.

#### Examples

In a recent study (Hodgson et al., 2019) systems biology approaches combining experimental and computational findings studied the mechanism by which TGFβ protects against pro-inflammatory responses and how this mechanism changes with age. Computational modeling revealed that two independent mechanisms were needed to explain the crosstalk between TGFβ and pro-inflammatory signaling pathways. Further insights into the mechanisms that cause TGFβ signaling to change from a protective to a detrimental pathway in cartilage with aging were provided. This systems biology approach suggests that the restoration of the protective role of TGFβ can be a potential therapy to prevent loss of cartilage in aging patients. Similar studies to identify other factors leading to cartilage breakdown and identification of curative and protective targets can be found in Proctor et al. (2014); Hui et al. (2016), and Kerkhofs et al. (2016).

### Multiscale Models of Joint Degeneration

**FIGURE 6:**
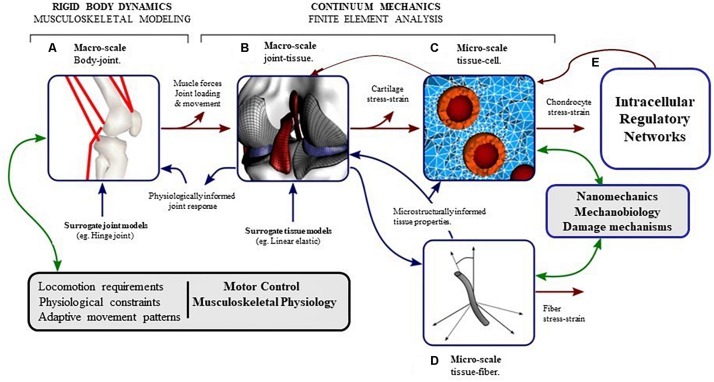
The pathway of mechanical signal from joint level to cellular (intra-cellular) level can be understood by developing computational models at different length scales which interact with each other. **(A)** Musculoskeletal (MSK) modeling approaches coupled with gait analysis can calculate the kinematics and the forces in the joint during locomotion. **(B)** Finite element analysis of the whole joint with inputs from MSK models can provide contact pressures and forces, and the macroscopic stresses and strains in the tissues. **(C)** Micro-scale finite element analysis of chondrocytes in the extracellular environment, with inputs from macroscopic finite element analysis of the tissue can produce deformations and stresses in the chondrocytes in their native environment. **(D)** Micro-scale fiber architecture and multiphase modeling can make the cellular-level models event more integrated. **(E)** The intracellular processes triggered by mechanical signals acting on the cell can be modeled by (Gene) Regulatory networks (modified from Halloran et al., 2012).

It is well-known from literature that moderate mechanical loading is essential to maintain cartilage homeostasis (Sah et al., 1989; Quinn et al., 1998; Bader et al., 2011) and excessive mechanical loading may trigger degeneration of cartilage. However, to understand the sequence by which mechanical signals are transferred from the joint level to the cellular level and how these signals trigger intracellular processes, multi-scale modeling is necessary (Halloran et al., 2012; Kapitanov et al., 2016). A brief framework of multiscale modeling for articular cartilage is shown in Figure 6.

#### Advantages and Limitations

Multi-scale modeling can be an important tool to evaluate the intricately linked mechanical states of the tissue and the cells, which can lead to the onset and progression of OA. A feed-forward post-processing approach (translating from the macro-scale tissue level to the micro-scale cell level) for multiscale modeling has the advantage of being less computationally intensive because the micro and macro scale models need to be solved only once for a particular loading scenario. However, in a uni-directional feed-forward approach, the reverse direction (i.e., translating from the micro to macro scale) is generally not implemented, which is important when tissue properties may change due to tissue damage or regeneration as in OA. This step would involve a computational homogenization approach like Geers et al. (2010) to infer the changes in the macro-scale tissue properties from the changes in micro-scale cartilage or bone properties due to intracellular processes. Such a homogenization procedure comes with specific computational challenges, a discussion of which is beyond the scope of this paper.

#### Examples

Multiscale modeling approaches allows to calculate (Sibole and Erdemir, 2012; Sibole et al., 2013) chondrocyte deformation due to physiological loading in the knee joint. In this post-processing approach, the macro-scale tissue level model was mechanically coupled to the micro-scale cellular model by passing the deformation gradient from the tissue level to the cell level. It was observed that micro-scale models calculated amplified deformations of the chondrocytes compared to those predicted at the macro-scale when simulating one body weight compressive loading at the tibio-femoral joint. Also, it was observed in Sibole et al. (2013) that first-order data passing (considering the first gradient of the deformation, i.e., deformation gradient tensor, as the link between macro and micro scales) between the different spatial scales appeared to be sufficient for simplified loading conditions. In a similar study using a post-processing approach (Tanska et al., 2015), a 3D multi-scale model was developed to compare chondrocyte and surrounding peri- and ECM responses under loading during gait in healthy and medial meniscectomy knee joints. Displacements and pore pressures at the nodes of a compartment level model (consisting only the medial compartment of knee joint) were used as boundary conditions for the cell level model. Medial meniscectomy *per se* was found not to alter chondrocyte deformations substantially during gait. However, abnormal joint loading following meniscectomy did expose the chondrocytes to higher magnitudes of fluid pressure and maximum principal strains. These changes might lead to loss of cell viability and contribute to the onset of OA.

A different multiscale framework was developed by Shim et al. (2011) and Fernandez et al. (2012), aiming at studying the initiation of OA at the bone-cartilage interface due to anterior cruciate ligament damage (ACLD). In the framework (shown in Figure 6), which was coded in CellML (Nickerson and Hunter, 2005), the cell level model involved a bone remodeling algorithm (Pivonka et al., 2008) based on the RANK–RANKL–OPG pathway that predicted the number of active osteoblasts (to deposit bone) and osteoclasts (to absorb bone). Similarly, a cartilage damage prediction model was used (based on the work of Nam et al., 2009), which quantitatively described the action of NF-κB signaling cascade under mechanical stimulation. Peak cartilage strains were used to excite IKK which activated the NF-κB pathway, thereby inducing a number of pro-inflammatory genes. It was observed using this model that ACLD resulted in subchondral bone thickening with a reduction in cartilage thickness. Also increased peak cartilage strains of the ACLD knee resulted in increase of inflammatory cytokines in the medial femoral condyle. One of the main contributions of this multi-scale approach was its ability to incorporate the effect of subchondral bone remodeling on cartilage inflammation.

### Data Driven Approaches

Artificial intelligence approaches such as machine learning (Du et al., 2018; Nelson et al., 2018; Jamshidi et al., 2019) constitute a different modeling approach to predict OA progression. The difference between these approaches to the others discussed in previous sections is that these approaches are fed purely with data and do not rely on underlying physics-based modeling approaches. In this section, the examples will focus on models based on patient reported outcome measures (PROMs). Other areas where data-driven approaches are commonly use are related to image processing and (G) RN inference.

#### Advantages and Limitations

The data-driven approaches are designed to deal with uncertainty and imprecision which are commonly present in clinical data sets such as those from OA studies. Generally, such prediction models are developed using input and output variables, with input variables consisting typically of baseline demographic or imaging data (Ashinsky et al., 2017; Du et al., 2018), and outcome variables consisting data related to the presence of knee OA as assessed by specific methods such as, Kellgren–Lawrence grade for classification of knee OA. The resulting model is then used to predict the outcome variables from a new data set (typically from independent patient data). The choice of the input variables is of great importance in this approach as it can affect the accuracy of the predictive models. Generally, known risk factors for OA are included in the list of input factors. The risk factors commonly include age, sex, and BMI. Some other suggestions for risk factors can be pharmacological treatments, genetic factors, varus or valgus misalignment of the knee, ethnicity, physical activity, etc. (Jamshidi et al., 2019). It is very important to identify the most important risk factors, as their inclusion will not only increase the accuracy of the prediction model but also reduce the number of redundant variables, thereby saving time and cost during the training phase of the model.

#### Examples

In Long et al. (2017), a model for the prediction of knee OA was developed. Kinetic variables from the hip and knee and the quality of life outcome score were combined to create a prediction model for predicting the risk of knee OA in post-traumatic individuals (with the least prediction error of 0.02). Imaging-based information incorporated in machine learning-based prediction models were found to improve their performance (Lazzarini et al., 2017). In Ashinsky et al. (2017), a disease classification model was developed using a machine learning algorithm to select features of articular cartilage from MRI (performed *in vivo*) indicative of OA progression. The selected features correlated with the Western Ontario and McMaster Universities Arthritis Index (WOMAC) score. The developed model predicted which patients would progress to having symptomatic OA with 75% accuracy.

## Verification, Validation, and Uncertainty Quantification of Computational Models

Establishing credibility of *in silico* models of biological processes follows a series of well-defined steps, summarized by the abbreviation VVUQ: verification, validation, and uncertainty quantification. The verification step refers to ensuring that the simulation outcomes correspond to the mathematical model. This means, amongst others, ensuring that no mistakes were made in the implementation of the model, that proper convergence studies were executed and that the solution proposed by the software is correct. In the validation step, one has to demonstrate that the simulation results correspond to the physical reality. This requires running simulations to predict conditions, within the model’s context of use, for which high-quality well-documented experimental data is available and performing the comparison between experimental and simulation results. For these first steps (verification and validation), a standard has recently been published for medical devices (ASME, 2018). This standard allows to assess the amount of V&V that is necessary to include in regulatory filing of a medical device, depending on the influence and consequence of the *in silico* model on the functioning of the device. Recently, it was elaborated how these same concepts can be applied to physiologically-based pharmacokinetic- models typically used in the context of drug development (Kuemmel et al., 2019). Finally, in the uncertainty quantification step, the aim is to obtain an understanding of the impact of the assumptions that were made while establishing the model and its parameters on the model outcome. Several excellent reviews describe the process of VVUQ in further detail for a variety of models and medical applications, see for instance (Steinman and Migliavacca, 2018; Parvinian et al., 2019) and references within. In the area of knee joint modeling, a recent paper by Erdemir et al. (2019) explicitly addresses issues such as reproducibility, model training, standards and regulatory affairs.

For validating musculoskeletal models, the first step is to compare the outputs of the developed model (which is calibrated from experimental data) with independent data sets. Most commonly, electromyography (EMG) data is compared to calculated muscle activation and force profiles. Less common is the comparison of the calculated joint contact forces against available experimental measures of joint loading from instrumented implants (Fregly et al., 2012). Ideally, the simulation is based on the corresponding experimental data for which the measured joint loading is available. Unique data sets are currently available in the biomechanics community for this purpose. Alternatively, musculoskeletal simulation outcomes (i.e., joint angles and moments, muscle activations and forces, muscle fiber and tendon velocities and internal joint loads) can be compared to the results obtained from previously validated published models, on the condition that identical input data (3D Motion capture and ground reaction force data) are used (Hicks et al., 2015).

Verification of FE models involves a wide range of approaches including mesh convergence studies, and has been extensively reported in literature (Jones and Wilcox, 2008; Erdemir et al., 2012). For FE models aimed at studying the sensitivity of general outcomes to some input parameters, a detailed validation may not be necessary. While developing FE models of subject specific *in vivo* cases, such as the knee or the hip joint, validation can be quite challenging due to unavailability of suitable experimental data (for example, measurement of joint contact forces *in vivo*) for both practical and ethical reasons. In such cases, partial validation of the *in vivo* model with experimental data for other specimens can be done. Non-invasive imaging techniques such as MRI can also be used to map the deformation of the cartilage, hence providing experimental data for validation. For validation of FE based degeneration models, one can use already established databases such as the OA initiative database, or use results from ongoing cohort studies. Since progression of OA is a long term phenomenon, in case of ongoing cohort studies, one has to wait for a long time to get the experimental data for the validation of prospective computational models. Some studies have used the OA database to compare their (retrospective) model predictions (Mononen et al., 2016; Liukkonen et al., 2017), which provided confidence in the predictive capability of the models.

Validation of multi-scale models invokes the need for experimental setups which can capture deformations or changes in the micro-scale cellular level due to changes in inputs in the macro-scale. Such multi-scale experimental setups can use multi-photon laser microscopy to measure chondrocyte deformations in both osteochondral grafts as well as intact joints (Fick et al., 2016; Moo et al., 2018). Parameters such as cell aspect ratio, volume and height and width are generally used to validate the simulation results with experiments (Sibole and Erdemir, 2012; Erdemir et al., 2015).

## Discussion and Conclusion

With a worldwide increase in OA prevalence, there is an urgent need of gaining a detailed understanding of the factors contributing to disease initiation, progression and remediation. The last decade, significant progress in the development of computational tools to study the complex biomechanical factors involved in OA was made, using computational models. Musculoskeletal and finite element modeling (Hosseini et al., 2014; Mononen et al., 2018) as well as (gene) regulatory networks (Shim et al., 2011) were used to study the initiation and progression of damage in the joint. This approach is widely regarded as one of the emerging approaches that can aid clinicians in planning timely interventions. Advanced imaging (Jadin et al., 2007; Xia et al., 2018; Honkanen et al., 2019) coupled with sophisticated computer modeling techniques can help in achieving this goal. In terms of modeling approaches, finite element models have been quite promising in predicting degeneration of different constituents of articular cartilage (Hosseini et al., 2014; Mononen et al., 2016) and the role of the subchondral bone in the disease process. However, there is still room for improvement in terms of the development of patient specific models. The primary bottleneck in developing patient specific *in silico* models of joint degeneration is the huge variability in joint geometry as well as mechanical properties of the corresponding tissues.

As evident from this review, algorithms for damage modeling of the different constituents of articular cartilage, i.e., the collagen fibrils, proteoglycans, and permeability of ground matrix have evolved from simpler damage models triggered by principal or deviatoric strains (Hosseini et al., 2014) to more complex damage models that take into account the cumulative effect of loading (Mononen et al., 2018) or using non-local damage theories (Párraga Quiroga et al., 2017). These algorithms are coupled with finite element models of the knee joint, and hence, are capable of predicting local cartilage degeneration due to biomechanical overloading of the joint. Another possible approach would be to add biomechanical factors into the statistical or machine learning-based predictive models. This would involve the use of data-driven methods with FE models and damage algorithms, which would provide information about the biomechanical factors in the model. However, this would require FE analysis of 100s of subjects which is a cumbersome and time consuming task. Furthermore, statistical or machine learning methods can possibly reduce the level of subject-specificity in terms of geometry and mechanics of the joint, by approaching a template-based analysis.

In this review paper, we also discussed the role of regulatory network models and their potential in predicting cell fate as a result of biomechanical and biochemical stimuli. To improve the robustness of such models, a complete description of the intracellular signaling pathways is needed thereby requiring unique experimental data that is increasingly made available from a range of microarray and RNA sequencing studies that describe the effect of mechanical loading on cell fate decisions. Also, if these regulatory networks were to be coupled with a larger multi-scale framework, the effect of abnormal joint loading on chondrocyte transcriptional activity and hence the phenotype change responsible for OA progression could ultimately be monitored. A major challenge in this field is to relate the changes in cell phenotype to macro level changes in the joint loading, which would require not only extensive experimentation to characterize the change in matrix constituents with change in cell phenotype, but also the application of computationally intensive homogenization methods for homogenization of micro-scale mechanical properties to the macro scale.

In conclusion, as described in this paper, a substantial number of computational tools are available that can be used to model joint degeneration in OA. In the future, efforts should be made to integrate the different modeling techniques into an open access more robust computational framework that should not only be efficient to predict OA progression but also easily allow individualized risk assessment for use in screening in clinical practice.

## Author Contributions

SM and LG conceived of the presented idea and the overview of the review. MN contributed to writing the subchondral bone section of the manuscript and SM wrote the rest of the manuscript. IJ and LG reviewed the manuscript and suggested improvements in the content.

## Conflict of Interest

The authors declare that the research was conducted in the absence of any commercial or financial relationships that could be construed as a potential conflict of interest.
